# The Non-Coding RNA Journal Club: Highlights on Recent Papers—5

**DOI:** 10.3390/ncrna3020021

**Published:** 2017-06-14

**Authors:** Cyrinne Achour, Baptiste Bogard, Florent Hubé, Sendurai A. Mani, Gaetano Santulli, Joseph H. Taube

**Affiliations:** 1 CNRS UMR7216, Epigenetics and Cell Fate, Université Paris Diderot, Sorbonne Paris Cité, UMR7216 Epigénétique et Destin Cellulaire, Bâtiment Lamarck B, Case Courrier 7042, 35 rue Hélène Brion, 75013 Paris, France; cyrinne.achour@gmail.com (C.A.); baptiste.bogard@univ-paris-diderot.fr (B.B.); florent.hube@univ-paris-diderot.fr (F.H.); 2 Department of Translational Molecular Pathology and 2Metastasis Research Center, The University of Texas MD Anderson Cancer Center, Houston, TX 77054, USA; smani@mdanderson.org (S.A.M.); Joseph_Taube@baylor.edu (J.H.T.); 3 College of Physicians & Surgeons, Columbia University Medical Center, 1150 St Nicholas Avenue, New York, NY 10032, USA; 4 Department of Advanced Biomedical Sciences, “Federico II” University, Naples 80131, Italy; gsantulli001@gmail.com

## 1. Introduction

We are delighted to share with you our **fifth** *Journal Club* and highlight some of the most interesting papers published recently. We hope to keep you up-to-date with non-coding RNA research outside of your area. The *Non-Coding RNA* Scientific Board wishes you an exciting and fruitful read.

## 2. A New Pathway for Small Non-Coding RNA

### Highlight by Cyrinne Achour and Florent Hubé

MicroRNAs (miRNAs) play a key role in the regulation of gene expression. In general, miRNAs are ~22 nucleotides that are generated from a two-step cleavage by Drosha in the nucleus and by Dicer in the cytoplasm. Then, miRNAs are recruited in the RNA-induced silencing complex (RISC) which contains Argonaute2 and guides the complex to the 3’ untranslated region (3’UTR) of the targeted mRNA.

Recently, a novel class of regulatory non-coding RNAs (ncRNAs), independent of Drosha and Dicer cleavage, has been described by Hansen et al [1]. These ncRNAs are named agotrons. Agotrons are processed from full short intron lariats to generate debranched introns and require Ago2 to be stabilized in the free 5’-ends. They have been characterized as short (~80–100 nt), stable and more GC-rich compared to ordinary introns. Particularly, the Pkd1 agotron, initially described as a mirtron—another unconventional miRNA originating from introns—has been shown to be specifically stable, conserved and acts like a miRNA to repress its target gene.

Thus, these uncommon ncRNAs have been identified but their sequence characteristics and structures have to be determined to better understand how they repress gene expression.

## 3. The Long Non-Coding RNA Encoding Proteins

### Highlight by Baptiste Bogard and Florent Hubé

Long non-coding RNAs (lncRNAs) are so called because of the lack of evidence for a protein coding function. Yet, an increasing number of lncRNAs hide information for short polypeptides, less than 100 amino acids, in addition to putative RNA-linked functions. For instance, the lncRNA LINC00961 was shown to encode a polypeptide SPAR (Small regulatory Polypeptide of Amino acid Response) that is conserved across species, with a transmembrane domain [2]. The characterization of SPAR’s interactome by mass spectrometry identified the v-ATPase–Regulator supercomplex as a target. SPAR was shown to inhibit the activation of the anti-proliferative mTOR (mammalian target of rapamycin) complex mTORC1 by preventing the release of its negative regulator subunit. Upon muscle injury, SPAR expression is decreased, enabling the activation of mTORC1 and hence, myofiber regeneration. Thus, SPAR is a peptide that fine tunes the regulation of the mTOR signaling pathway to facilitate context-specific cellular responses.

This study adds additional evidence, if necessary, that the non-coding definition of lncRNAs is somewhat fuzzy, and that we may now have to set new limits and refer to protein- or peptide-(non)-coding RNAs.

## 4. Alternative Splicing Can Both Produce Non-Coding and Coding RNAs in the Same Gene

### Highlight by Baptiste Bogard and Florent Hubé

Alternative splicing is a remarkable strategy employed by eukaryotic cells to increase and diversify the transcriptional output of a gene, offering the possibility to produce various isoforms. These events result, at least in part, from variations in the processivity of the RNA polymerase II (RNAPII) and in the transcription elongation rate that, under some physiopathological conditions, can favor the production of one or the other isoforms. Williamson et al. [3] observed that the elongation rate of the RNAPII is reduced after UV irradiation, and document alternative splicing events of several genes, particularly of the alternative last exon (ALE).

We noticed the case of ASCC3 transcripts, whose shorter isoform is preferentially transcribed after irradiation at the expense of the longer isoform. The short ASCC3 isoform is a functional non-coding RNA that is important for the recovery of transcription after UV-irradiation. In contrast, the longer ASCC3 isoform produces a protein known to participate in transcriptional repression. This antagonistic function of two isoforms, coding and non-coding, produced from the same locus is very reminiscent of what was shown for the founding member of the family of bifunctional RNAs, SRA (Steroid Receptor RNA Activator) and its cognate protein SRAP, with antagonistic functions in muscle differentiation.

## 5. The *MAYA* lncRNA Links Hippo Signaling and ROR1 Activation to Bone Metastasis

### Highlight by Joseph H. Taube and Sendurai A. Mani

Hippo signaling leading to cytoplasmic sequestration of YAP is well-established as a regulator of proliferation, tumorigenesis and metastasis. However, ROR1, an orphan receptor tyrosine kinase, is less well understood, despite its overexpression in multiple types of cancer. In a tour-de-force, Li and Wang et al. [4] show that the lncRNA, *MAYA* (MST1/2-antagonizing for YAP activation), unites these two signaling molecules to activate Hippo signaling and regulate bone metastasis. Previously uncharacterized, the authors show that *MAYA* functions in the cytoplasm as a scaffold to facilitate methylation of MST1 by NSUN6, which is then recognized by a combination of *MAYA* and the protein LLGL2. Activation of this pathway is facilitated by neuregulin binding to heterodimerized ROR1 and HER3. Elegant biochemistry firmly establishes the binding capacity of *MAYA* for multiple simultaneous interacting proteins while in vivo assays demonstrate the necessity of *MAYA* for breast cancer cells to metastasize to bone. As breast cancer patients with activated HER3 exhibit unfavorable progression-free survival, therapies designed to block this pathway merit further investigation [4].

## 6. Linking Inflammation and Beta Cell Death: The Functional Role of MicroRNAs

### Highlight by Gaetano Santulli

In an interesting paper published in *Diabetes*, Decio Eizirik and colleagues demonstrate that specific microRNAs (miRNAs) are functionally involved in the control of inflammation-induced apoptosis in human pancreatic beta cells [5]. The authors show that three miRNAs (miR-23a-3p, miR-23b-3p, and miR-149-5p) were down-regulated in human islets exposed to IL-1 and IFN-γ. These miRNAs are involved in the modulation of key pro-apoptotic proteins. Therefore, these findings provide new mechanistic insights into the multifaceted dialogue between the immune system and pancreatic beta cells, with major implication in the pathophysiology of type 1 diabetes mellitus.
